# *In silico* Identification of Key Factors Driving the Response of Muscle Sensory Neurons to Noxious Stimuli

**DOI:** 10.3389/fnins.2021.719735

**Published:** 2021-09-10

**Authors:** Sridevi Nagaraja, Luis F. Queme, Megan C. Hofmann, Shivendra G. Tewari, Michael P. Jankowski, Jaques Reifman

**Affiliations:** ^1^Department of Defense Biotechnology High Performance Computing Software Applications Institute, Telemedicine and Advanced Technology Research Center, United States Army Medical Research and Development Command, Fort Detrick, MD, United States; ^2^The Henry M. Jackson Foundation for the Advancement of Military Medicine, Inc., Bethesda, MD, United States; ^3^Department of Anesthesia, Division of Pain Management, Cincinnati Children’s Hospital Medical Center, Cincinnati, OH, United States; ^4^Department of Pediatrics, University of Cincinnati, College of Medicine, Cincinnati, OH, United States

**Keywords:** musculoskeletal pain, nociceptor, ion channels, computational analysis, action potential

## Abstract

Nociceptive nerve endings embedded in muscle tissue transduce peripheral noxious stimuli into an electrical signal [i.e., an action potential (AP)] to initiate pain sensations. A major contributor to nociception from the muscles is mechanosensation. However, due to the heterogeneity in the expression of proteins, such as ion channels, pumps, and exchangers, on muscle nociceptors, we currently do not know the relative contributions of different proteins and signaling molecules to the neuronal response due to mechanical stimuli. In this study, we employed an integrated approach combining a customized experimental study in mice with a computational model to identify key proteins that regulate mechanical nociception in muscles. First, using newly collected data from somatosensory recordings in mouse hindpaw muscles, we developed and then validated a computational model of a mechanosensitive mouse muscle nociceptor. Next, by performing global sensitivity analyses that simulated thousands of nociceptors, we identified three ion channels (among the 17 modeled transmembrane proteins and four endoplasmic reticulum proteins) as potential regulators of the nociceptor response to mechanical forces in both the innocuous and noxious range. Moreover, we found that simulating single knockouts of any of the three ion channels, delayed rectifier voltage-gated K^+^ channel (Kv1.1) or mechanosensitive channels Piezo2 or TRPA1, considerably altered the excitability of the nociceptor (i.e., each knockout increased or decreased the number of triggered APs compared to when all channels were present). These results suggest that altering expression of the gene encoding Kv1.1, Piezo2, or TRPA1 might regulate the response of mechanosensitive muscle nociceptors.

## Introduction

Acute pain is a natural response to musculoskeletal injury. While the sensation of pain in response to injury involves neurons in both the peripheral and central nervous systems (PNS and CNS), the first step in the initiation of a pain response is the activation of the nerve endings of specialized sensory neurons that innervate musculoskeletal tissue, known as nociceptors. Nociceptors in muscle tissue respond to noxious peripheral stimuli, such as mechanical forces (distinct from light contact), extreme heat or cold temperatures, and high concentrations of metabolites produced by contracting muscles, by transducing them into electrical signals (Light et al., 2008; Ross et al., 2014; de Moraes et al., 2017). The electrical signal in a nociceptor is generated by depolarization of its membrane potential, usually referred to as the generator potential. Once the generator potential reaches a sufficient threshold, it results in the firing of an action potential (AP) (Pak et al., 2018). The threshold at which APs are fired and their firing rate encode information about the presence and severity of peripheral stimuli. APs travel along slowly conducting unmyelinated (C) or thinly myelinated (Aδ) axons of nociceptors with small- or medium-diameter cell bodies in the dorsal root ganglion (DRG) to the CNS and subsequently to the brain, where they are processed and may lead to a pain sensation (Pak et al., 2018).

The threshold at which an AP is generated as well as its height, width, and frequency are determined by many classes of transmembrane proteins, including ion channels, ion pumps, and exchangers of four main ions (i.e., K^+^, Na^+^, Ca^2+^, and Cl^–^), present on the nociceptor (Julius and Basbaum, 2001; Dubin and Patapoutian, 2010). However, we still do not fully understand the individual contributions of different transmembrane proteins to AP generation in response to peripheral stimuli and transmission of these signals by the nociceptors. The vast heterogeneity in transmembrane protein expression on muscle nociceptors and the different mechanisms by which these proteins recognize and respond to specific noxious stimuli (Julius and Basbaum, 2001; Woolf and Ma, 2007) make it challenging to identify the key proteins that regulate pain signaling. Yet, identification of such proteins is essential to help improve our understanding of how acute pain is initiated as well as the changes in acute pain signaling that can lead to pathological pain scenarios, such as chronic pain, where both the expression and function of certain proteins at the nerve endings and DRGs are altered (Gold and Gebhart, 2010). Eventually, this knowledge will be useful in the delineation of efficacious treatments to alleviate pain.

Despite the considerable progress made toward understanding nociceptor signaling in animal models and pre-clinical studies, to date, there are only a few effective non-opioid therapeutic interventions for pain management (Woolf, 2020). One of the main reasons for this is that newly identified molecular targets fail to demonstrate efficacy in clinical trials. For example, until recently, Nav1.7 channels expressed in many nociceptors, including those in muscle tissue, were a promising target for treating pain based on the observation that animals with mutations involving Nav1.7 function loss exhibit a strong decrease in pain (Bennett et al., 2019). However, clinical trials for Nav1.7-selective channel blockers were not successful (McDonnell et al., 2018). Experimental investigations of muscle nociceptors are especially challenging because their free nerve endings are hard to access given their small diameters and intricate branching within the several layers of muscle tissue (Mense, 2010). Thus, the majority of the electrophysiological investigations of individual membrane proteins are performed on nociceptor cell bodies in the DRGs. Moreover, compared to other afferent neurons, muscle nociceptors exhibit unique AP firing properties due to the large diversity in their expression of different transmembrane proteins both at the DRG and the nerve endings. Finally, none of the transmembrane proteins and signaling molecules work in isolation. Therefore, it is important to quantify the contributions of different transmembrane proteins to AP responses, both individually and relative to the observed changes in the expression and function of other proteins.

Computational modeling approaches can complement traditional experimentation in the search for key proteins and signaling molecules that regulate nociceptor signaling. For instance, mathematical models can be used to estimate the contribution of specific proteins to the neuronal response by predicting the effects of blocking or overexpressing each protein in a milieu of nociceptors (involving wide-ranging expressions of different transmembrane proteins) in a systematic and time-efficient manner. In fact, previous computational models of pain signaling in nociceptive neurons yielded insights into the roles of specific ion channels. For example, Mandge and Manchanda (2018) used a computational model of a rat bladder DRG neuron to demonstrate that increased conductance of rectifying small-conductance calcium-activated potassium channels can increase AP firing in those neurons. However, with a few exceptions, existing models have been developed to represent medium- or large-diameter DRG neurons, and have been focused on DRGs of neurons that innervate tissues other than muscle, such as DRGs in the gastrointestinal tract (Chambers et al., 2014), rat bladder (Mandge and Manchanda, 2018), or other non-specific DRG neurons (Luscher et al., 1994; Amir and Devor, 2003; Baker, 2005; Tigerholm et al., 2014; Sundt et al., 2015). Moreover, most models do not incorporate the endoplasmic reticulum (ER) calcium release and the uptake mechanisms that affect intracellular Ca^2+^ signaling and Ca^2+^-activated transmembrane proteins (Verkhratsky, 2005). Given the high variability exhibited by neurons depending on the physiological tissue they innervate (de Moraes et al., 2017), computational models must incorporate the transmembrane mechanisms that are pertinent to pain signaling in a given neuron type. Thus, to understand the pain-signaling mechanisms in musculoskeletal tissue, we need to develop a computational model based on experimental data specific to muscle nociceptors.

In this study, we primarily aimed to identify key transmembrane proteins that regulate the response to mechanical forces in mouse muscle nociceptors. To this end, we developed a mathematical model that incorporates the major transmembrane protein mechanisms as well as the ER mechanisms known to be present in mechanosensitive muscle nociceptors, i.e., those that specifically respond to mechanical stimuli. The model represents 14 ion channels (including three known mechanosensitive channels, i.e., Piezo2, TREK-1, and TRPA1), two ion pumps, one ion exchanger, and four ER membrane mechanisms. To customize and make our model specific to mouse muscle nociceptors, we performed new *ex vivo* experiments on sensory afferent neurons in the hindpaw muscles of wild-type C57BL/6J mice. We used a subset of these data to calibrate our model parameters such that our simulated AP response to an external current and mechanical forces matched the corresponding experimental data. Then, we used the remaining data to validate our model results by comparing the predictions of the AP response to an external current and mechanical forces with the corresponding experimental data, and showed that our model captured the AP responses of the mouse muscle nociceptors reasonably well. Upon model validation, we performed global sensitivity analysis (GSA) by simulating the responses to mechanical forces in 50,000 nociceptors to quantify the contribution of the different modeled proteins to AP generation. From this analysis, we identified three ion channels (i.e., Kv1.1, Piezo2, and TRPA1) as key contributors to APs generated in response to mechanical force. Finally, we used the model to investigate the specific effects of separately knocking out each of the three ion channels and found that the knockout (KO) of Kv1.1 or TRPA1 increased AP generation (compared to when all channels are present), suggesting that alterations in the expression and function of these two channels might contribute to nociceptor sensitization. In contrast, the KO of Piezo2 primarily decreased AP generation, suggesting that manipulation of this channel might be a potential strategy to reduce neuronal excitability. Thus, we used our validated model of a mouse muscle mechanical nociceptor to generate experimentally testable hypotheses regarding the key transmembrane proteins that regulate the AP response to peripheral mechanical stimuli.

## Materials and Methods

### Experimental Methodology

#### Animals

We used 20 adult male wild-type C57BL/6J mice 3–6 weeks of age. The mice were housed in a climate-controlled barrier facility with 12-h light/dark housing and *ad libitum* access to food and water. All procedures were approved by the Institutional Animal Care and Use Committee (IACUC) at Cincinnati Children’s Hospital Medical Center and the Animal Care and Use Review Office (ACURO) of the Department of Defense under AAALAC approved practices.

#### Electrophysiological Recordings

We used the *ex vivo* mouse hindpaw muscle/tibial nerve/DRG/spinal cord recording preparation previously developed by our group (Dourson et al., 2021) to obtain electrophysiological recordings of individual muscle afferent neurons. Briefly, we excised the spinal cord and the right hindpaw from mice deeply anesthetized with 90 mg/kg ketamine and 10 mg/kg xylazine, and placed them in a bath filled with a solution of oxygenated artificial cerebrospinal fluid (aCSF) at ∼10°C. Next, we dissected the hindpaw muscles, tibial nerve, and DRG neurons at spinal cord vertebrae L1-L6 together with their corresponding segments and transferred them to a separate recording chamber filled with oxygenated aCSF. Finally, we slowly warmed the ice-cold aCSF recording solution to 32°C, before recording the responses of neurons located in L3 and L4 DRGs. We chose to record from the L3 and L4 DRGs because they are the primary source of muscle afferent fibers contained in the tibial nerve that innervate the flexor digitorum brevis (FDB) muscles, our target muscle to locate receptive fields.

To locate the afferent neurons innervating the hindpaw muscles, we first placed a suction electrode onto the side of the tibial nerve, and then applied orthograde electrical search stimuli (0.4–2 mA, 1 ms in duration at 0.5 Hz) to identify neurons with axons in the tibial nerve. We then used a concentric bipolar electrode to identify receptive fields (RFs) of the electrically identified neurons in the FDB muscle. After determining that the recorded cells contained a RF in the FDB, we applied a mechanical stimulus (Force, 1-100 mN; recovery time between stimuli, 20–30 s) using an increasing series of calibrated von Frey filaments (Dourson et al., 2021). To characterize the response properties of the recorded afferents, we then stimulated the RFs with cold (∼1°C) and hot (∼52°C) saline, followed by a low-concentration metabolite mixture (15 mM lactate, 1 mM ATP, pH 7.0). Finally, after a wash out and resting period, we stimulated the RFs with a high-concentration mixture of the same metabolites (50 mM lactate, 5 mM ATP, pH 6.6). Whenever possible, we repeated the application of mechanical and thermal stimuli to determine any acute metabolite-mediated sensitization of the mechanically sensitive afferent subpopulations. We recorded the response properties of the DRG neurons to these stimuli *via* sharp intracellular quartz microelectrodes (impedance > 150 Ω) containing 5% neurobiotin in 1 M potassium acetate. We used the response latency to the electrical stimulation delivered by the suction electrode to calculate the conduction velocity (CV) and to classify the afferents into one of two groups, III (CV > 1.5 m/s) or IV (CV ≤ 1.5 m/s). We subsequently cataloged the recorded neurons based on their response properties to the different stimuli applied. We obtained electrophysiological recordings from 33 individual neurons, from which, after offline analysis, we identified neurons that were sensitive to mechanical force stimuli (*N* = 20) and only used data from those neurons to develop and validate the computational model.

### Computational Model

**FIGURE 1:**
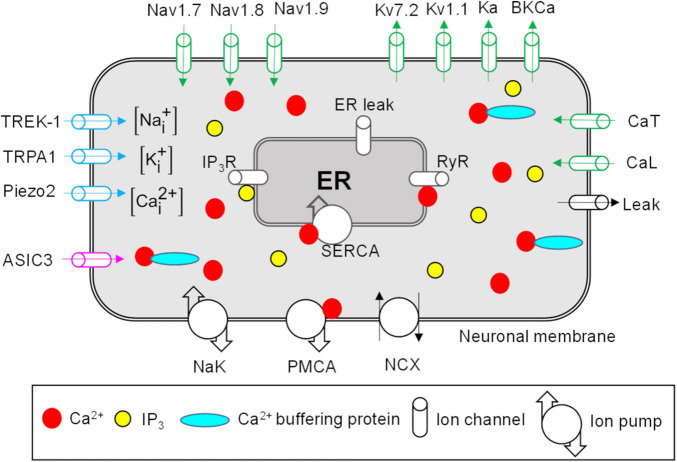
Neuronal membrane and endoplasmic reticulum (ER) proteins represented in the model. Shown are all the modeled neuronal transmembrane proteins and ER membrane proteins. Voltage-gated ion channels are shown in green, mechanosensitive ion channels in blue, and the pH-sensitive ion channel in pink. Ion pumps and exchangers as well as a passive leak ion channel are shown in black. The ER was modeled as a compartment inside the neuronal nerve ending. The modeled ER mechanisms included inositol trisphosphate (IP_3_) receptors (IP_3_R), ryanodine receptors (RyR), SERCA pump, and ER leak channel (shown in gray). The arrows on ion channels indicate the direction of flow of the ions conducted by those channels. The intracellular compartment of the nociceptor’s nerve ending consisted of Na^+^, K^+^, Ca^2+^, Ca^2+^-binding buffer proteins, and IP_3_. PMCA, Ca^2+^-ATPase pump; NCX, Na^+^-Ca^2+^ ion exchanger; NaK, Na^+^-K^+^-ATPase; SERCA, sarco/endoplasmic reticulum Ca^2+^-ATPase pump.

We developed a computational model that represents the afferent nerve ending of a generic mechanosensitive mouse muscle nociceptor. The model accounts for the kinetics of the known active and passive mechanisms in the nociceptor’s neuronal membrane and ER, as well as its intracellular Ca^2+^ dynamics (Figure 1). These mechanisms are discussed below.

#### Neuronal Membrane Mechanisms

In our model, we included the descriptions of 17 transmembrane proteins, including 14 ion channels, two pumps, and an exchanger, that are present on the membrane of muscle nociceptors and are essential for their signaling (Woolf and Ma, 2007; Mense, 2010). The specific modeled proteins are described below.

##### Voltage-Gated Ion Channels

We modeled nine voltage-gated ion channels, including three Na^+^ channels, four K^+^ channels, and two Ca^2+^ channels (Figure 1, green ion channels). Voltage-gated Na^+^ channels, such as Nav1.7, Nav1.8, and Nav1.9 (Blair and Bean, 2002), and voltage-gated K^+^ channels, such as Kv7.2, Kv1.1, and A-type (Ka) (Barkai et al., 2017; Du et al., 2018; D’Adamo et al., 2020), are expressed in small DRGs and provide depolarizing and repolarizing currents to the membrane potential during APs, respectively. Therefore, we included the description of these six channels in our model. Furthermore, large-conductance Ca^2+^-activated K^+^ (BKCa) channels have been shown to contribute to AP duration and firing in small DRGs (Lu et al., 2014). Hence, we included these channels in our model. Finally, we modeled one low-voltage-gated (CaT) and one high-voltage-gated (CaL) Ca^2+^ channel (Lee, 2013). CaT is activated at membrane potentials close to the resting membrane potential (RMP) and is involved in its maintenance. We adapted the mathematical equations and model parameters describing the above-mentioned ion channels (except CaT) from Mandge and Manchanda (2018), and represented the CaT channels using Boltzmann equations (Sigg, 2014), with the equation parameters derived from the work of McCallum et al. (2003).

##### Passive Leak Channel

We modeled a passive leak channel (Figure 1, black ion channel) to incorporate currents through the voltage-independent ion channels, such as TRAAK, that are expressed in small DRGs (La et al., 2011) and contribute to the maintenance of the RMP. We adapted the mathematical description of this channel from Mandge and Manchanda (2018).

##### Mechanosensitive Ion Channels

We modeled three mechanosensitive ion channels that conducted at least one of the three modeled ions (i.e., Na^+^, K^+^, and Ca^2+^) (Figure 1, blue ion channels). Mechanosensitive channels Piezo1 and 2 are expressed in mouse muscle tissue (Coste et al., 2010) and, specifically, Piezo2 has been shown to function as a mechanotransducer in murine skin (Coste et al., 2010). Furthermore, cation channels from the transient receptor potential family (TRPA1) and the two-pore K^+^ channel family (TREK-1) are activated by mechanical stimulation and are present in small-diameter DRGs (Alloui et al., 2006; Vilceanu and Stucky, 2010; Acosta et al., 2014). In our model, we described Piezo2 using the equations and parameters provided in Fujita (2014) and described the TRPA1 and TREK-1 channels using Boltzmann equations, where the parameter values were mechanical forces. We derived these from experimental data obtained from isolated thoracolumbar DRGs for TRPA1 (Brierley et al., 2011) and transiently transfected COS cells for TREK-1 (Patel et al., 1998).

##### Acid-Sensing Ion Channels

We modeled one Na^+^-conducting ASIC3 channel (Figure 1, pink ion channel). Various isomers of acid-sensing ion channel (ASIC), such as ASIC1a and b, ASIC2a and b, and ASIC3, which are activated by pH changes in the extracellular fluid, are expressed in skeletal muscle afferents (Boscardin et al., 2016; Ortega-Ramirez et al., 2017). In the model, we described the ASIC3 channel using a Boltzmann equation, where we derived the pH-dependent equation parameters from Abdelhamid and Sluka (2015).

##### Ion Pumps and Exchangers

We modeled two ion pumps [i.e., Na^+^-K^+^-ATPase (NaK) and Ca^2+^-ATPase (PMCA)] and the ion exchanger Na^+^-Ca^2+^ (NCX). Active transmembrane ion pumps and exchangers are present on the muscle nociceptors and use energy from ATP hydrolysis to transport ions across the neuronal membrane against their concentration gradient (Hamada et al., 2003; Gover et al., 2007; Kuroda et al., 2013). In our model, we adapted the equations describing the two ion pumps and the ion exchanger from Kapela et al. (2008) and Mandge and Manchanda (2018), respectively.

#### Intracellular Ca^2+^ Dynamics

The key ionic components inside the nociceptor are the intracellular concentrations of Na^+^, K^+^, and Ca^2+^. In addition, the intracellular compartment of the nociceptor also contains the ER and diffusible second messengers, such as inositol trisphosphate (IP_3_) and Ca^2+^ buffering proteins (Figure 1). Intracellular Ca^2+^ and ER Ca^2+^ in small DRGs are regulated by ER Ca^2+^ release and uptake mechanisms (described in the next section below) (Verkhratsky, 2002) as well as Ca^2+^ binding to the buffering proteins (Matthews and Dietrich, 2015). Accounting for Ca^2+^ binding to buffering proteins is essential for determining the availability of free intracellular Ca^2+^ ions that can alter the function of other ion channels and pumps in both the neuronal (e.g., BKCa and PMCA) and ER [e.g., ryanodine receptors (RyRs)] membranes. In our model, we adapted the description of Ca^2+^ binding with buffering proteins from Mandge and Manchanda (2018), where we modified some of the parameters to match the experimental data of intracellular and ER Ca^2+^ changes in Verkhratsky (2002).

#### ER Membrane Mechanisms

The ER can occupy ∼10% of the neuronal volume and acts as a reservoir of Ca^2+^ inside the neuron (Figure 1). The ER membrane contains IP_3_ receptors (IP_3_Rs) and RyRs (Figure 1, gray ion channels). IP_3_Rs are activated by intracellular IP_3_ and Ca^2+^, and RyRs are activated by Ca^2+^ present near the ER membrane. The ER membrane also contains SERCA, an active ATPase pump, which helps replenish the ER Ca^2+^ lost *via* opening of the IP_3_Rs and RyRs by actively transporting Ca^2+^ into the ER. SERCA is an important regulator of intracellular Ca^2+^ transients in small DRGs (Verkhratsky, 2002). Finally, the ER contains passive leak channels that maintain its basal Ca^2+^ concentration. In our model, we adapted the kinetic descriptions for the IP_3_Rs, RyRs, ER leak channels, and SERCA from Mandge and Manchanda (2018), and modified some of the parameter values describing these mechanisms to match the experimental data of intracellular and ER Ca^2+^ changes reported by Verkhratsky (2002).

#### Model Simulations, Inputs, and Outputs

Our model is a coupled system of 30 ordinary differential equations (ODEs). Each equation represents one model variable, where a variable represents activation or inactivation factors of 13 ion channels; the intracellular concentration of K^+^, Na^+^, Ca^2+^, and IP_3_; the ER Ca^2+^ concentration; and the membrane potential (*V*_*m*_). Table 1 provides a list of the model variables, their descriptions, and initial values. Using the lumped Hodgkin-Huxley-type formalism (Hodgkin and Huxley, 1952), we calculated the changes in *V*_*m*_ at a given time point from the changes in the currents through all the neuronal transmembrane proteins described above as follows:

**TABLE 1:** Model variable names, descriptions, and initial values.

Variable name	Description	Initial value	Variable name	Description	Initial value
Nav1.8_*m*_	Activation constant of the voltage-gated Nav1.8 channel	0	TREK_*m*_	Activation constant of the two-pore TREK-1 K^+^ channel	0
Nav1.8_*h*_	Inactivation constant of the voltage-gated Nav1.8 channel	1	BKCa_*n*_	Activation constant of the large-conductance Ca^2+^-activated K^+^ channel	0
Nav1.7_*m*_	Activation constant of the voltage-gated Nav1.7 channel	0	Ka_*n*_	Activation constant of the M-type K^+^ channel	0
Nav1.7_*h*_	Inactivation constant of the voltage-gated Nav1.7 channel	1	Ka_*hfast*_	Fast inactivation constant of the M-type K^+^ channel	1
Nav1.9_*m*_	Activation constant of the voltage-gated Nav1.9 channel	0	Ka_*hslow*_	Slow inactivation constant of the M-type K^+^ channel	1
Nav1.9_*h*_	Inactivation constant of the voltage-gated Nav1.9 channel	1	Kv7_*n*_	Activation constant of the voltage-gated Kv7.2 channel	0
Piezo_*m*_	Fast activation constant of the Piezo2 channel	0	Kv1.1_*n*_	Activation constant of the delayed rectifier Kv1.1 channel	0
Piezo_*h*_	Inactivation variable of the Piezo2 channel	1	CaL_*m*_	Activation constant of the L-type voltage-gated Ca^2+^ channel	0
ASIC3_*m*_	Activation constant of the ASIC3 channel	0	CaL_*h*_	Inactivation constant of the L-type voltage-gated Ca^2+^ channel	1
ASIC3_*h*_	Inactivation constant of the ASIC3 channel	1	CaT_*m*_	Activation constant of the T-type voltage-gated Ca^2+^ channel	0
TRPA1_*m*_	Activation constant of the TRPA1 channel	0	CaT_*h*_	Inactivation constant of the T-type voltage-gated Ca^2+^ channel	1
TRPA1_*h*_	Inactivation constant of the TRPA1 channel	1	*V*_*m*_	Membrane potential	−55 mV
$\left[{\text{Na}}_{\text{i}}^{+}\right]$	Intracellular Na^+^ concentration	14 mM	$\left[{\text{K}}_{\text{i}}^{+}\right]$	Intracellular K^+^ concentration	140 mM
$\left[{\text{Ca}}_{\text{i}}^{2+}\right]$	Intracellular free calcium ion concentration	5 × 10^–5^ mM	$\left[{\text{Ca}}_{\text{ER}}^{2+}\right]$	Calcium concentration in the endoplasmic reticulum	0.25 mM
IP_3_	Intracellular inositol trisphosphate concentration	1 × 10^–5^ mM	hIP_3_	Activation constant of the IP_3_ receptor	0.667

(1) $$\begin{array}{l} \frac{\text{d}V_{\text{m}}}{dt}=(I_{Nav1.8}+I_{Nav1.9}+I_{Nav1.7}+I_{Piezo}+I_{ASIC3}+I_{TRPA1}\\ \;+I_{TREK}+I_{Kv7.2}+I_{Kv1.1}+I_{BKCa}+I_{Ka}+I_{Kleak}+I_{CaT}\\ \;+I_{CaL}+I_{PMCA}+I_{NaK}+I_{NCX}+I_{Stim})/C_{m} \end{array}$$

where *C*_*m*_ denotes the membrane capacitance, *I* represents the current through the different transmembrane proteins (described by the subscripts), and *I*_*Stim*_ denotes an external stimulation current. We used 87 parameters to describe all the modeled mechanisms (neuronal and ER membrane). Table 2 provides a list of the model parameter numbers (used to keep track of the parameters in our simulations), name, value, description, unit, and the source of the computational or experimental study from which we adapted or derived their values, respectively. We modified a subset of the model parameters (designated as “modified” in Table 2) to match baseline AP properties, such as AP amplitude, AP height, and AP duration, obtained from our experimental study (see section “Model Calibration and Validation” below). In all simulations, we maintained the extracellular concentrations of K^+^, Na ^+^, and Ca^2+^, the volume of the nociceptor nerve ending, and its membrane capacitance (*C*_*m*_) at a constant value. Table 3 shows the values of the model parameters that we kept constant during the sensitivity analysis. We provide the ODEs and other equations describing all the modeled mechanisms, as well as the Nernst potentials and ionic balances for the intracellular concentrations of Na^+^, K^+^, and Ca^2+^ in the Supplementary Information.

**TABLE 2:** Model parameter number (used in the model), name, description, value, units, and sources.

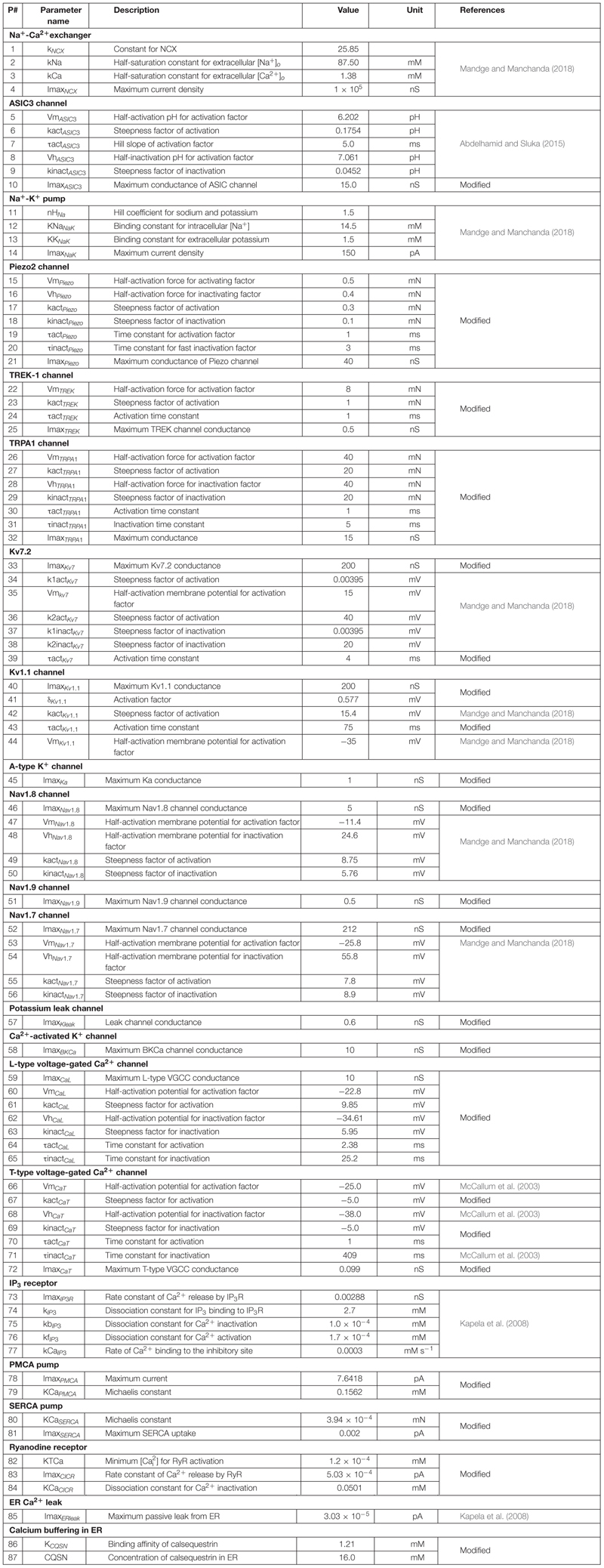

**TABLE 3:** Parameters held at constant values during sensitivity analysis.

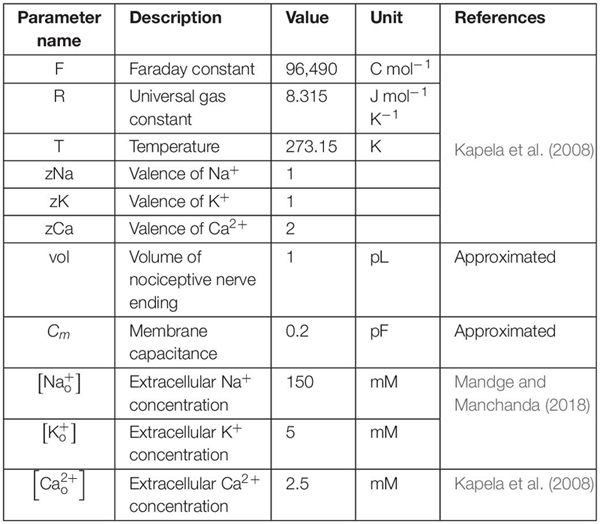

To drive the model and replicate our experimental protocol, we provided as inputs a rectangular pulse of 80 pA external current followed by a series of six rectangular pulses with mechanical forces of 0.7, 4, 10, 20, 40, and 100 mN. We applied each pulse for a period of 10 s, with a 20-s period between them. We applied the first pulse (i.e., the external current) at the simulation time point of 40 s, for a total simulation time of 250 s. At the end of each simulation, our model provided a 250-s time course for each of the 30 model variables. In all our computational analyses, we focused on the membrane potential time course, from which we calculated the total number of APs generated following the application of each pulse of mechanical force. We defined an AP as a membrane potential spike of at least 15 mV from its resting value. We used the MATLAB function FINDPEAKS to identify the APs and to record their height and width as well as the simulation time points at which they were generated. We performed all computations using MATLAB R2018b (MathWorks, Natick, MA, United States) and solved the system of 30 ODEs using the MATLAB solver ODE15s, with default tolerance levels.

### Model Calibration and Validation

#### Model Calibration

**FIGURE 2:**
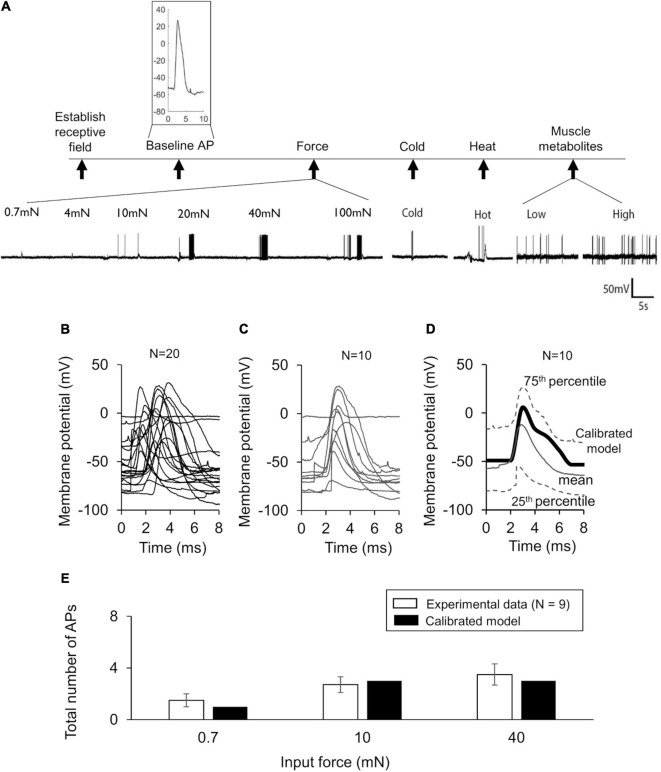
Model calibration using experimental data. **(A)** Shown are representative membrane potential recordings from a single afferent neuron in wild-type C57BL/6J mouse hindpaw in response to an electrical stimulation [used to generate the baseline action potential (AP)], six mechanical forces, hot and cold temperatures, and muscle metabolites (black vertical arrows). Inset shows a 10-ms trace of the baseline AP extracted from the recording. **(B)** Shown are the 10-ms traces extracted from the electrophysiological recordings of 20 independent afferent neuron membrane potential recordings. We divided these traces into two sets of 10 neurons each. **(C,D)** Shown are the baseline AP dataset used for model calibration and the model calibration results, respectively. The solid gray line and the upper and lower dashed gray lines represent the mean baseline AP and the 25th and 75th percentile of the calibration data, respectively. The thick black line represents the model simulation of the AP following an external current stimulus of 80 pA. **(E)** Shown are the results of the model calibration to the AP response after application of mechanical forces. Open bars represent the mean and one SE of the number of APs generated by seven mechanically sensitive neurons in response to three different mechanical forces. The number of neurons that responded to a specific mechanical force differed across the three applied forces: *N* = 2 for 0.7 mN, *N* = 6 for 10 mN, and *N* = 9 for 40 mN. Solid bars represent the corresponding APs in the simulation.

**FIGURE 3:**
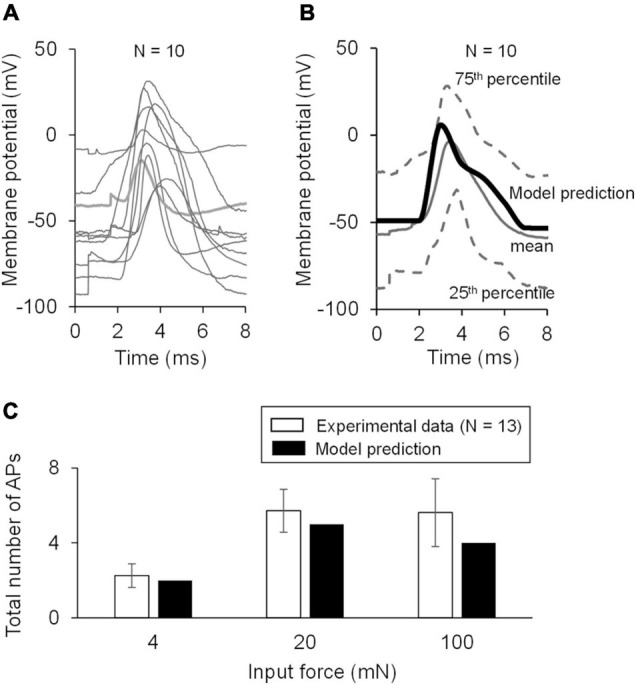
Model validation using experimental data. **(A)** Shown are the baseline APs from the dataset used for model validation. **(B)** Shown is the comparison of the model prediction of the AP generated in response to an external current of 80 pA (thick black line) with the mean baseline AP of the validation data (solid gray line). Our model prediction was within the 25th and 75th percentile (low and high dashed gray lines, respectively) of the validation data. **(C)** Shown is the comparison of the model predictions of the APs generated in response to forces of 4, 20, and 100 mN with the corresponding experimental data. The number of neurons that responded to a specific mechanical force differed across the three applied forces: *N* = 4 for 4 mN, *N* = 6 for 20 mN, and *N* = 12 for 100 mN. Open bars represent the mean and one SE of the number of APs generated by mechanically sensitive neurons. Solid bars represent the corresponding APs in the simulation.

To make our model specific to the mouse muscle nociceptor, we calibrated it using our experimental data in two steps. First, we calibrated the model so that it matched the baseline AP generated by the neurons in response to an electrical stimulation. To this end, we extracted 10-ms traces of the baseline APs from the raw electrophysiological recordings of the 20 mechanically sensitive neurons (see section “Experimental Methodology” above) (Figures 2A,B). Then, we randomly divided the traces into two sets of 10 traces each, where we used one of the sets for model calibration (Figure 2C) and the other for model validation (Figure 3A). To calibrate the model parameters, we modified the values of a subset of 11 the model’s 87 parameters associated with Nav1.7, Nav1.8, Nav1.9, Kv1.1, Kv7.2, Ka, BKCa, NaK, and PMCA (designated as “modified” in Table 2) such that the simulated AP response to a 80 pA current fell within the 25th and 75th percentile of the corresponding calibration data (Figure 2D). Specifically, we tried to match the AP amplitude, AP height, and AP duration between the model simulation and the mean baseline AP of the calibration dataset. Next, we calibrated the model so that it matched the AP response of the neurons to mechanical forces. To this end, we calculated the number of APs generated by the mechanically sensitive neurons following the application of each of the six mechanical forces and divided these data into two sets, where we used the data for the 0.7, 10, and 40 mN forces (Figure 2E, open bars) for model calibration and those for the 4, 20, and 100 mN forces for model validation. To perform the calibration, we modified the values of a subset of 23 model parameters associated with Piezo2, TREK-1, TRPA1, Kv1.1, and Kv7.2 (designated as “modified” in Table 2) such that the number of simulated APs in response to 0.7, 10, and 40 mN were within one standard error (SE) of the corresponding experimental data. We used the Akaike information criterion (AIC) (Cavanaugh and Neath, 2019) to ensure that we only modified the minimum number of parameter values necessary to capture the experimental data, while avoiding overfitting the model. We provide a brief description of the AIC analysis and its results in the Supplementary Table S1. We defined the model’s “nominal parameter set” as the final parameter values obtained after performing the two calibration procedures.

#### Model Validation

To validate our model, we first compared our model prediction (using the nominal parameter set) of the AP response induced by a current stimulus of 80 pA with the mean baseline AP obtained from the validation subset comprised of 10 mechanically sensitive neurons not used for calibrating the model (Figure 3A). Next, we compared our model predictions of the number of APs generated in response to mechanical forces of 4, 20, and 100 mN to the corresponding experimental data (Figure 3C).

### Global Sensitivity Analysis

First, we performed a local sensitivity analysis (LSA) to assess the model’s robustness and remove any non-essential interactions, as previously described (Nagaraja et al., 2014). In this analysis, we varied the model parameters near their nominal values (±1%). Next, to quantify the contributions of the various transmembrane and ER proteins to the AP response and identify its key regulators, we performed two types of GSA: a partial rank correlation coefficient (PRCC) analysis and an extended LSA. For each of these analyses, we simulated 50,000 distinct nociceptive signaling conditions. First, we generated 50,000 unique parameter sets by randomly selecting parameter values from a fourfold range (twofold in each direction) around the nominal parameter values. This random sampling attempted to capture the known heterogeneity in the expression of the various proteins at different nerve endings of muscle nociceptors as well as the variability in the conductance, activation, and inactivation gating factors of the same membrane proteins under different stimuli (Gold and Gebhart, 2010). To generate the random parameter sets, we used Latin hypercube sampling (MATLAB function LHSDESIGN) (Nagaraja et al., 2014). Next, we performed simulations using the 50,000 parameter sets, where we drove each simulation using an 80 pA current pulse followed by a sequence of six increasing mechanical forces (i.e., 0.7, 4, 10, 20, 40, and 100 mN). We stopped and eliminated the simulations that did not reach the 250 s of the membrane potential time course within 30 s of computing (wall-clock) time or that required time steps smaller than 1 × 10^–12^ s. We used this lack of convergence in the simulations to flag parameter sets that resulted in non-physiological kinetic behavior. Accordingly, we only used the simulations that ran to completion to calculate the number of APs generated, and their durations and heights following the application of mechanical forces. We used the number of APs generated in response to each mechanical force stimulus as our primary output.

#### PRCC Analysis

For this analysis, we calculated the Spearman’s PRCCs and their associated *p* values between the primary output and each of the 87 model parameter values. The values of the PRCC vary between −1 and +1, where large absolute values reflect high impact of a particular model parameter on the model output (i.e., the number of APs). The sign of the PRCC indicates the positive or negative directionality of the correlation between the model parameter and the output. A PRCC with a *p* value < 0.01 indicated that it was significantly different from zero. Upon completion of this analysis, we obtained six sets (one for each applied force) of 87 PRCC values along with their associated *p* values.

#### Extended LSA

For this analysis, we calculated the relative local sensitivity indices s*_*j*_*(*t*) of the *j*^*th*^ parameter at different simulation time points *t* using the ratio of derivatives (Nagaraja et al., 2014):

(2) $$s_{j}\,\left(t\right)=\frac{\text{d}\,V_{\text{m}}}{V_{\text{m}}}/\frac{{\text{dP}}_{j}}{{\text{P}}_{j}}$$

where *V*_*m*_ denotes the membrane potential variable and P*_*j*_* denotes model parameter *j* (of the model’s 87 parameters). Each parameter was individually perturbed by ± 1% of its nominal value, and the derivative was approximated using the second-order central finite difference formula. These sensitivities reflect the magnitude of the relative change in the membrane potential induced by a local (i.e., a small) change of a given model parameter. In the subset of the 50,000 parameter sets whose simulations successfully converged, we calculated these at a single time point following the application of each of the six mechanical forces. We selected the six time points at which we had observed the AP peak in response to the six forces in the simulation using the nominal parameter set. Upon completion of this analysis, we obtained six sets (one for each applied force) of 87 sensitivity indices for each simulation.

### Identification of Key Transmembrane Proteins That Regulate Nociceptor AP Response

We utilized the results from the GSAs to identify key transmembrane proteins that could regulate AP generation in a muscle nociceptor. Using the results from the PRCC analysis, we divided the set of PRCCs calculated for each applied mechanical force into groups using a *k*-means clustering algorithm (MATLAB function KMEANS) (Nagaraja et al., 2017), to identify model parameters in the top group comprised of the highest absolute PRCC values. We identified key AP regulators as the model parameters with PRCCs in this group that also had *p* values ≤ 0.01. Using the results from the extended LSA, for each applied mechanical force, we first ranked the absolute values of the 87 sensitivities in descending order in each simulation. Next, we calculated the percentage of the simulations (out of the total simulations that ran successfully) in which each model parameter’s sensitivity ranked the highest, and designated the parameters within the top four highest percentage as key for AP regulation. Finally, we identified the model parameters regarded as key for AP regulation in both the PRCC and extended LSA calculations, and labeled the transmembrane proteins dependent on these parameters as key proteins for AP-response regulation.

#### *In silico* Ion Channel KO Analysis

In order to investigate the impact of the model-identified key proteins on the APs generated by muscle nociceptors, we performed simulations in which we separately knocked out each key transmembrane protein. To simulate such a KO, we set the current in Eq. (1) corresponding to that protein to zero. First, we simulated KOs using a model with the nominal parameter set. Then, to verify that we could reproduce the effects of the different protein KOs (e.g., an increase in AP generation), we repeated the KO simulation of each key protein using 10,000 parameter sets randomly selected from the group of successful simulations in the GSA. Similar to the GSA, we stopped simulations that did not reach the 250 s of the membrane potential time course within 120 s of computing (wall-clock) time to flag parameter sets where a protein KO resulted in non-physiological kinetic behavior. Of the KO simulations that converged successfully, we calculated the number of simulations in which the KO of a specific key protein increased or decreased the number of APs generated compared to when all channels were present as well as the average increase or decrease in the number of APs generated in those simulations.

## Results

### Model Captures AP Response to Electrical and Mechanical Stimulation

In our experiments, we recorded the electrophysiological responses of 33 (13 C-fibers and 20 Aδ-fibers) afferent neurons innervating the mouse hindpaw muscles to four different types of stimuli: electrical, mechanical force, hot and cold temperatures, and muscle metabolites (Figure 2A). We classified the 33 neurons as Type IV (C-) or Type III (Aδ-) fiber afferents based on their conduction velocity [C: 0.51 m/s (SD = 0.1) and Aδ: 12.15 m/s (SD = 6.5)]. Of these 33 neurons, 20 were mechanically sensitive (i.e., generated an AP in response to at least one of the six applied mechanical forces). These neurons comprised of both C- (*N* = 7) and Aδ-afferents (*N* = 13), however, we did not find any statistically significant differences in the baseline AP characteristics (i.e., RMP, AP width, and overshoot) or in the response to mechanical stimuli (i.e., mean threshold and AP firing rate) between these two groups of neurons (Supplementary Table S2). The neurons in both groups constituted a mix of polymodal and unimodal phenotypes sensitive to mechanical, thermal, and chemical stimuli (AMCH-Met and CMCH-Met); mechanical and cold stimuli (AMC and CMC); and only mechanical stimuli (AM and CM). We used the data from all of these 20 neurons (Figure 2B) to calibrate and validate the model’s response to an electrical stimulus. The mean RMP of these 20 neurons was −54.31 (SD = 21.05) mV. However, of the 20 mechanically sensitive neurons, five only responded to mechanical stimulation after exposure to high concentrations of muscle metabolites in the bath. Because our model currently does not account for neuronal sensitization or for the neuronal response to chemical stimuli, we only considered data from the 15 neurons, comprised of six C-fiber neurons and nine Aδ-fiber neurons, that were sensitive to mechanical stimuli prior to any metabolite exposure for calibration and validation of the model’s response to mechanical stimuli. In addition, because some of these neurons responded to low, non-noxious force values (0.7–4 mN) and may not necessarily be nociceptors, but rather mechanoreceptors, we re-classified each of the 15 neurons as either mechanical nociceptors or mechanoreceptors based on their ability to encode the severity of the applied force in their AP response. We then assessed whether there was a difference in their mechanical responses. We did not find any statistically significant differences in the mechanical threshold or AP firing between the two groups (Supplementary Table S3) and, hence, used all 15 neurons together for calibration and validation of the model’s mechanical response.

We first calibrated the model to the mean baseline AP response elicited by an electrical stimulation of the 10 neurons in the calibration set (Figure 2C). This resulted in the simulated AP response to a current stimulus of 80 pA to fall within the 25th and 75th percentile of the baseline AP for the calibration data (Figure 2D). Next, we calibrated the model to match the number of APs generated by nine of the 15 mechanically sensitive neurons in response to mechanical forces of 0.7, 10, and 40 mN, leading to results that fell within one SE of the experimental data (Figure 2E). We designated the final set of model parameter values obtained after these two calibration procedures as the nominal parameter set. Finally, to assess the stability of the model, we performed a LSA using the nominal parameter set (see section “Global Sensitivity Analysis”) and found that the membrane potential was not drastically sensitive (sensitivity indices > 100) to any one of the model’s 87 parameters, suggesting that the model was stable and robust to small perturbations (±1%) of its nominal values.

To validate our model, we first predicted the neuronal response to an applied current of 80 pA using the nominal parameter set and compared this prediction to the mean baseline AP data (Figure 3B, solid black line vs. solid gray line) for the 10 neurons in the validation set not used for model calibration (Figure 3A). Our AP prediction was within the 25th and 75th percentile of the validation data (Figure 3B, thick black line). Next, using the nominal parameter set, we predicted the neuronal response to mechanical forces of 4, 20, and 100 mN and compared the model-predicted number of APs in response to each of the three forces to the corresponding experimental data. Our predictions were within one SE of these validation data (Figure 3C). To summarize, we performed a new study and collected data from mechanically sensitive C- and Aδ-fiber mouse muscle nociceptors to develop and validate a computational model of a mechanosensitive muscle nociceptor.

### Key Ion Channels for AP Response Regulation

To identify the transmembrane proteins that strongly regulated the AP response (specifically the number of APs generated) across many different nociceptor signaling conditions, we used two distinct, yet complementary, GSAs (PRCC and extended LSA). Of the 50,000 simulations performed for each of these analyses, 43,967 ran successfully in the PRCC analysis while 40,934 ran successfully in the extended LSA (see section “Global Sensitivity Analysis”).

**FIGURE 4:**
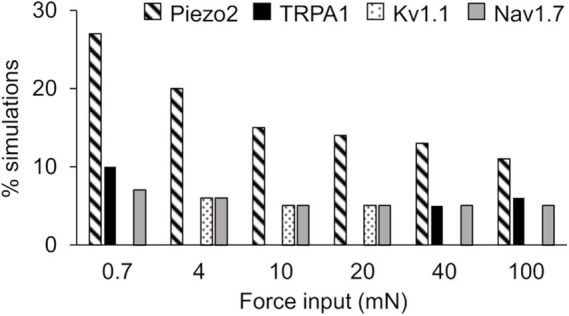
Extended local sensitivity analysis (LSA) identified key ion channels for action potential regulation. Shown is the percentage of the 40,934 simulations (see section “Key Ion Channels for AP Response Regulation”) for which a local change (±1%) in the model parameters associated with Piezo2 (diagonally striped), TRPA1 (solid black), Kv1.1 (dotted), and Nav1.7 (solid gray) induced the highest sensitivity in the membrane potential after the application of mechanical forces ranging from 0.7 to 100 mN.

In the extended LSA, we identified model parameters associated with the 17 membrane proteins whose local changes (±1%) yielded the highest sensitivity in the membrane potential after the application of a mechanical force, for all of the six mechanical forces. Across all forces, changes in the model parameters associated with the mechanosensitive channel Piezo2 and the voltage-gated Na^+^ channel Nav1.7 yielded the highest sensitivities in ∼11–27% (Figure 4, diagonally striped) and ∼5–7% (Figure 4, solid gray), respectively, of the total 40,934 simulations. For forces of 4, 10, and 20 mN, changes in the parameters associated with Kv1.1 yielded the highest sensitivities in ∼5–6% (Figure 4, dotted) of the simulations. For forces of 0.7, 40, and 100 mN, changes in the parameters associated with TRPA1 yielded the highest sensitivities in ∼5–10% (Figure 4, solid black) of the simulations. The model parameters associated with other membrane proteins yielded the highest sensitivities in fewer than 5% of the simulations and are not shown here. Thus, among the 40,934 distinct nociceptor simulations, the parameters associated with Piezo2, Nav1.7, Kv1.1, and TRPA1 consistently and strongly affected the membrane potential in response to a wide range (0.7–100 mN) of applied forces.

**FIGURE 5:**
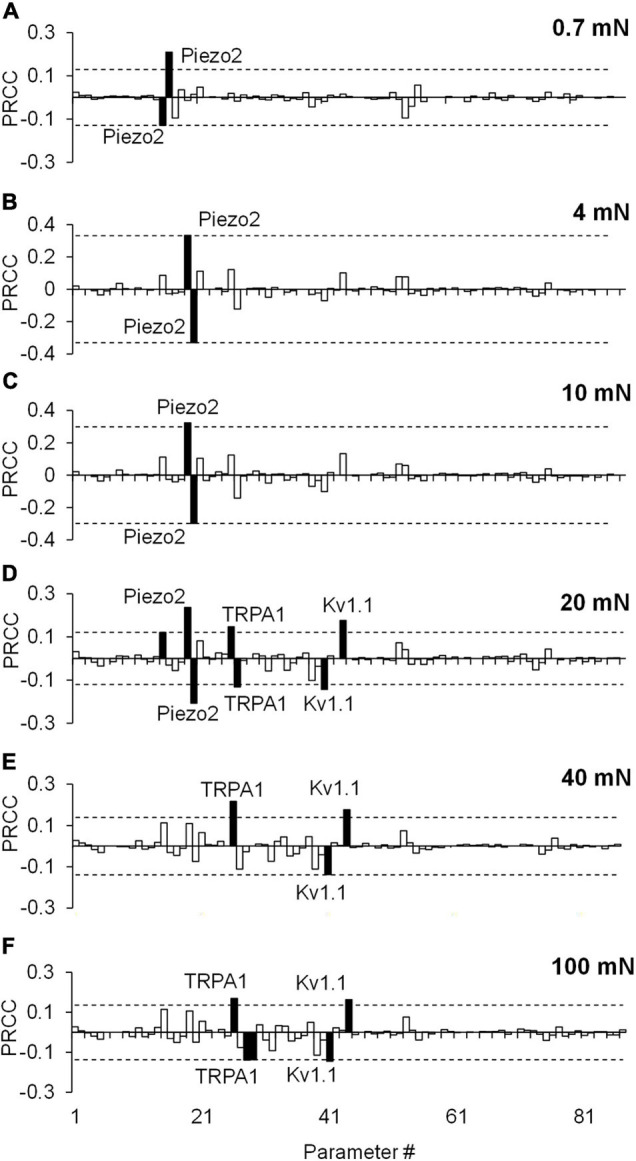
Partial rank correlation coefficient (PRCC) analysis identified key ion channels for action potential (AP) regulation. The bars show the PRCCs of the 87 model parameters with the number of APs generated calculated from 43,967 simulations (see section “Key Ion Channels for AP Response Regulation”) after the application of a mechanical force of **(A)** 0.7 mN, **(B)** 4 mN, **(C)** 10 mN, **(D)** 20 mN, **(E)** 40 mN, and **(F)** 100 mN. The PRCCs that were above their respective thresholds (dashed horizontal lines) and were statistically significant (i.e., those for which *p* < 0.01) are indicated by solid black bars, and the labels of the bars show the ion channels that these parameters describe in the model.

Similar to the results of the extended LSA, the PRCC analysis results showed that, for the majority of the applied forces, the model parameters associated with Kv1.1, Piezo2, and TRPA1 channels (Figure 5, solid black bars) yielded high and statistically significant correlations (*p* < 0.01) with the number of generated APs. Similar to the results of the extended LSA, model parameters associated with Piezo2 were strongly correlated to the AP firing for low forces (0.7–10 mN) while those associated with TRPA1 were strongly correlated to high forces (20–100 mN). To summarize, we used two types of GSA and identified three ion channels (i.e., Kv1.1, Piezo2, and TRPA1) from the 17 modeled transmembrane proteins as key regulators of AP generation, whose modifications could alter the neuronal response to mechanical forces in muscle nociceptors. Even though some of the specific functions of the model parameters related to these key ion channels (e.g., the modulation of the time constant of activation of the Kv1.1 channel) may be experimentally difficult or unfeasible to manipulate, we may still be able to perform experiments involving the addition of antibodies and ion channel inhibitors or use genetically modified animals (Chi and Nicol, 2007; Kerstein et al., 2009; Du et al., 2018) to achieve the desired AP regulation. To identify potential (and feasible) experiments that could clarify the role of these key channels, we performed simulations where we separately knocked out each of the three channels and investigated their effect on AP generation.

### *In silico* KO Analysis of the Model-Identified Key Proteins

**FIGURE 6:**
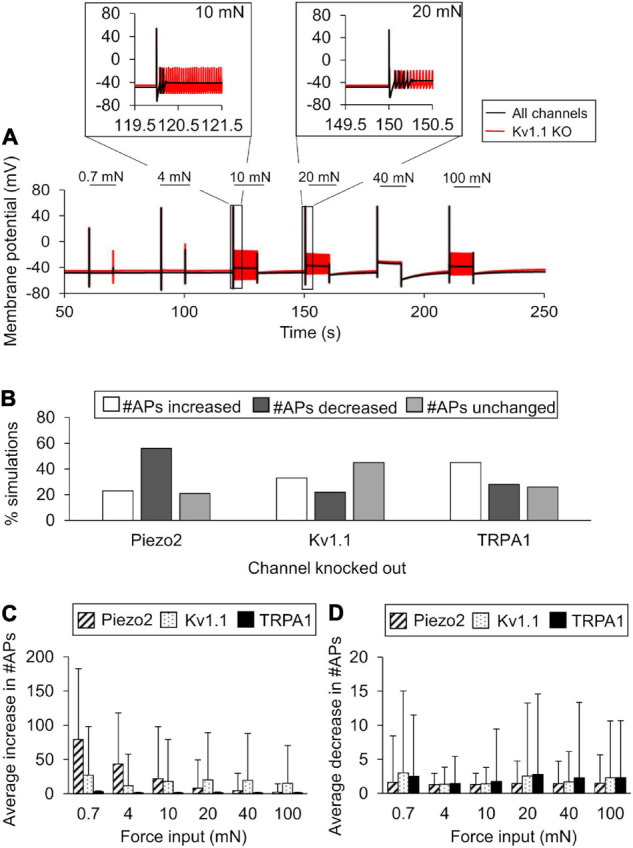
*In silico* ion channel knockout (KO) analysis identified channels that might contribute to AP generation. We simulated the KO of the three model-identified key ion channels using the nominal parameter set and 10,000 randomly selected parameter sets (see Ion channel KO simulations in section “Materials and Methods”). **(A)** Shown are the model-predicted 250-s time courses of the membrane potential simulated using the nominal parameter set with all channels present (solid black line) and with Kv1.1 KO (red line) in response to mechanical forces of 0.7, 4, 10, 20, 40, and 100 mN (represented by horizontal black lines at the top). Insets show the action potential (AP) response to 10 and 20 mN. **(B)** Shown are the percentages of simulations (out of 9,900) in which the KO of Kv1.1, Piezo2, or TRPA1 resulted in an increase (open bars), decrease (dark gray bars), or no change (light gray bars) in the number of APs generated compared to when all channels were present in response to at least one of the six mechanical forces, as well as the means and one SD of the increases **(C)** and decreases **(D)** in the number of APs for each channel KO in those simulations.

We performed KO simulations for each of the three key ion channels using models based on both the nominal parameter set as well as 10,000 parameter sets randomly selected from the group of successfully completed simulations in the extended LSA. In the simulations with the nominal parameter set, only Kv1.1 KO demonstrated an increase in APs generated in response to 4, 10, 20, and 100 mN forces (Figure 6A, black line vs. red line), while both TRPA1 KO and Piezo2 KO reduced the number of APs generated (data not shown). Of the 10,000 KO simulations we performed for each of the three channels, 9,900 completed successfully, and we used the results from those simulations for further analysis (see section “*In silico* Ion Channel KO Analysis”). First, we calculated the number of simulations (out of 9,900) in which the number of APs increased, decreased, or remained unchanged after each channel KO compared to when all channels were present. For Kv1.1 KO, AP generation increased in ∼33% of the total simulations, decreased in ∼22%, and remained unchanged in ∼45% (Figure 6B). Similar to Kv1.1, TRPA1 KO increased AP generation in the majority of the simulations, i.e., the number of APs generated increased in ∼45% of the TRPA1 KO simulations, decreased in ∼28%, and remained unchanged in ∼26% (Figure 6B). In contrast to Kv1.1 and TRPA1, Piezo2 KO decreased AP generation in the majority of the simulations, i.e., the number of APs generated decreased in ∼56% of the Piezo2 KO simulations, increased in ∼23%, and remained unchanged in ∼21% (Figure 6B). Next, to determine the magnitude of the increase or decrease in AP generation caused by each channel KO, we calculated the mean and SD of the increase or decrease in the number of APs from the corresponding subsets of simulations. The means of the increase in APs for Piezo2 and Kv1.1 KOs (calculated from individual mean increase values for all six forces) were ∼27 (SD = 30) and ∼19 (SD = 5), respectively (Figure 6C, diagonally striped and dotted), while the means of the decrease in APs for the respective channel KOs were ∼1.4 (SD = 0.1) and ∼2 (SD = 0.6) (Figure 6D, diagonally striped and dotted, respectively). Thus, when Piezo2 and Kv1.1 KO resulted in an increase in AP generation, we observed that the effect was ∼10 times stronger than when their KO resulted in a decrease in AP generation. On the other hand, the means of the increase and decrease in APs in the TRPA1 KO simulations were of the same order of magnitude and <5 APs on average [mean increase ∼1.6 (SD = 0.6) and mean decrease 2.1 (SD = 0.5)] (Figures 6C,D, solid black). Thus, our results suggest that while the KO of either Kv1.1 or TRPA1 mainly increased AP generation, the magnitude of the increase was higher with Kv1.1 KO, suggesting that Kv1.1 might be crucial for nociceptor sensitization. Moreover, Piezo2 KO primarily decreased AP generation and, therefore, might be a potential target for reducing the excitability of muscle nociceptors in response to mechanical forces.

## Discussion

Musculoskeletal pain is a widespread problem with few non-opioid efficacious treatment options (Woolf, 2020). Muscle tissue is innervated by several different nociceptor subtypes that transduce noxious stimuli into an electrical pain signal *via* the action of several transmembrane proteins and signaling molecules. Both the large number of different transmembrane proteins involved in this process as well as the vast heterogeneity in their expression and activity complicate the identification of key regulators of nociception in muscle tissue. In this study, we used both experimental and computational methods to identify key proteins regulating a muscle nociceptor’s response to mechanical force stimuli. We developed a computational model of a mechanosensitive mouse muscle nociceptor that accounted for the activity of 17 neuronal membrane proteins and four ER membrane proteins, and calibrated and validated the model using new experimental data collected from wild-type C57BL/6J mice. Our computational model successfully captured the nociceptor’s response to electrical and mechanical force stimuli (Figures 2, 3). Using the validated model, we simulated 50,000 unique nociceptors to account for the observed heterogeneity in protein expression on these nociceptors and identified three ion channels (i.e., Kv1.1, Piezo2, and TRPA1) that strongly regulated the AP generation in response to mechanical forces. Moreover, by separately simulating KOs of each of these three channels, we determined that KO of Kv1.1 in muscle nociceptors increased AP firing and might be important for nociceptor sensitization.

### Challenges in Determining the Contribution of Different Transmembrane Proteins to AP Generation

Muscle nociceptors, like cutaneous nociceptors, respond to a wide variety of stimuli (heat, cold, mechanical force, and chemicals) (Julius and Basbaum, 2001; Gold and Gebhart, 2010). Even though biophysical studies on the DRGs of nociceptive neurons have identified distinct proteins that can transduce these specific stimuli, it is challenging to establish these transduction mechanisms *in vivo* because such observations do not always correspond with those made *in vitro*. For example, the presence of a known acute-cold transducer such as TRPA1 (Story et al., 2003) does not always warrant cold sensitivity *in vivo*, which at times depends on the presence of tissue injury or inflammation (del Camino et al., 2010). In addition, TRPA1 knockout reduces cold sensitivity in some but not all studies (Kwan et al., 2006; Karashima et al., 2009; del Camino et al., 2010). Moreover, transduction mechanisms of noxious stimuli *in vivo* can also involve indirect mechanisms, such as the release of inflammatory mediators from surrounding cells in the tissue that change the relative density and distribution of membrane proteins, which ultimately alter the neuronal response. Such combinatorial effects among transmembrane proteins make it particularly challenging to unravel the role of individual proteins, especially when multiple distinct proteins act in parallel to accomplish similar functions (Gold and Gebhart, 2010). We attempted to address these challenges by simulating 50,000 muscle nociceptors representing a heterogeneity in protein expression and activity that mimic the numerous plausible protein combinations *in vivo* and subsequently evaluating the contribution of each protein to mechanical nociception under distinct conditions.

Ultimately, in addition to inconsistencies in observations within animal studies, there is always the question of translatability of the nociceptive response across species. While a few studies have investigated the similarities and differences between the molecular profiles of human and mouse nociceptors (Mogil, 2019), understanding how nociceptive mechanisms translate from one species to another requires direct comparisons between them. Although advancement in technologies like transcriptomics and microneurography have allowed for a deeper *in vitro* and *in vivo* investigation of human nociceptors, such opportunities are limited due to the scale and diversity of human nociceptor populations (Middleton et al., 2021). Until we can reliably assess human nociceptors, animal models remain a necessary tool to gain a causal and mechanistic understanding of the role of different proteins involved in nociception.

### Key Mechanosensitive Ion Channels for AP Response

Among the many different types of transmembrane proteins (i.e., ion channels, pumps, and exchangers) that contribute to pain signaling, ion channels have been studied extensively as molecular targets to alleviate pain and reduce nociceptor sensitization (Woolf and Ma, 2007; Benarroch, 2015; Woolf, 2020). In fact, our computational analysis of 50,000 simulated nociceptors identified three ion channels as key for regulating responses to mechanical forces, including the mechanosensitive ion channels Piezo2 and TRPA1 and as well as the voltage-gated K^+^ channel Kv1.1 (Figures 4, 5).

The mechanosensitive properties of Piezo2 were identified recently (Coste et al., 2010), and there is evidence showing that Piezo2 KO in mice could suppress their mechanosensitivity and acute pain response (Murthy et al., 2018; Szczot et al., 2018). In fact, even in our Piezo2 KO simulations, we observed that the number of APs generated decreased in a majority (i.e., 56%) of them, indicating a reduction in mechanosensitivity (Figure 6B). Surprisingly, in a relatively smaller subset of simulations (23%), Piezo2 KO resulted in a sizeable increase in the number of APs generated (∼27 APs on average), especially for forces between 0.7 and 20 mN (Figures 6B,C). Because this result was unexpected, we further analyzed the values of the model parameters associated with membrane proteins other than Piezo2 to investigate whether any were considerably different between the two subsets of simulations where Piezo2 KO increased or decreased AP generation. Upon comparing the means of the 87 model parameter values in the two subsets, we found one such model parameter representing the inactivation of the voltage-gated Na^+^ channel Nav1.7 whose mean value was ∼5% lower in the subset where the AP generation increased compared to where it decreased (data not shown). This finding suggests that if Nav1.7 inactivation is suppressed in addition to Piezo2 KO, any membrane potential depolarization induced by the activation of other mechanosensitive channels, such as TRPA1 and TREK-1, might be augmented due to the slow Nav1.7 inactivation, leading to an increase in AP generation even in the absence of Piezo2 channel current. Overall, our results suggest that Piezo2 KO primarily reduced AP generation in muscle nociceptors and that Piezo2 could be a potential target to regulate acute pain response in muscles. However, depending on the activity of certain channels, such as Nav1.7, Piezo KO may sometimes lead to an increase in AP firing.

TRPA1, expressed in both C- and Aδ-fiber neurons, is involved in the transduction of chemical and mechanical stimuli (Vilceanu and Stucky, 2010). In fact, TRPA1 deletion in cutaneous C-fiber nociceptors in mice reduced AP firing by 50% in response to a wide range of mechanical forces, while in Aδ-fiber nociceptors such a response was only observed for high values of mechanical forces (>100 mN) (Kwan et al., 2009). Another study, where TRPA1 activity (instead of its expression) was blocked by a HC-030031 inhibitor, also showed a decrease in the responsiveness of mouse skin C-fiber nociceptors to the application of large mechanical forces (>40 mN) (Kerstein et al., 2009). Surprisingly, we found that AP generation increased more often in TRPA1 KO simulations (45%) than it decreased (28%) (Figure 6B). However, in either case, the effect was minor (average increase ∼1.6 APs, average decrease ∼3 APs) (Figures 6C,D, solid black). Similar to the Piezo2 KO simulations, we investigated whether the values of the parameters associated with membrane proteins other than TRPA1 were considerably different between the two subsets of TRPA1 KO simulations and could perhaps explain the unexpected result of the increase in AP generation in the majority (51%) of these simulations. We found two model parameters, one associated with the half-activation force value and the other with the steepness factor of activation of Piezo2 channels, that decreased by ∼10% and increased by ∼6%, respectively, in the subset where TRPA1 KO increased AP generation (data not shown). Thus, in those simulations, the activation of Piezo2 channels required a lower force and the channels opened at a faster rate than the other subset, which might compensate for the absence of TRPA1 channels and even increase AP firing. Thus, our results suggest that the role of TRPA1 in muscle nociceptors is not as discernable as in their cutaneous counterparts, and further investigation of muscle nociceptors in TRPA1-deficient mice is needed to determine if targeting TRPA1 alone is sufficient to regulate the nociceptor’s excitability to mechanical stimuli.

### Key Voltage-Gated Potassium Channel for AP Response

Voltage-gated potassium channels regulate RMP, AP shape and firing properties, and overall neuronal excitability (Benarroch, 2015). In fact, our model-identified key regulator, Kv1.1 channel, has been previously studied as a possible molecular target for treating different neuronal pathologies (Tsantoulas and McMahon, 2014). Kv1.1 channels limit the duration of APs by remaining open during depolarization and promote the onset of repolarization. Given the crucial function of Kv1.1 during the development of an AP, we were not surprised to observe a considerable increase in AP firing (up to ∼100 APs) in response to a wide range of mechanical forces (1–100 mN) in 33% of our Kv1.1 KO simulations (Figures 6B,C). Indeed, selective blocking of Kv1.1 has been shown to increase AP firing in rat sensory DRG neurons (Chi and Nicol, 2007) and, based on our results, it is likely that blocking Kv1.1 increases AP firing in mouse muscle nociceptors as well. While it is clear that Kv1.1 can reliably regulate APs, current studies focus on achieving selective blockade of Kv1.1 in specific neurons located in tissues of interest. Given its ubiquitous presence in several locations in the body and known contributions to AP generation in other excitable cells, e.g., cardiomyocytes, Kv1.1 manipulation can lead to extreme clinical outcomes, such as sudden death (D’Adamo et al., 2020). Thus, the challenge of future studies is to selectively block or overexpress Kv1.1 solely in muscle nociceptors, which will allow us to test its viability as a molecular target for regulating pain response in muscle tissue.

### Assumptions and Limitations

Our computational model has several limitations arising from simplifying assumptions required to capture the complex signaling of transmembrane proteins in muscle nociceptors. First, we recorded the AP responses to various mechanical stimuli (applied to the mouse hindpaw) at the cell body of mouse sensory neurons located in the DRG. By doing so, we assumed that the AP generated at the nerve endings of the nociceptor travels in an intact manner to the DRG. Second, we used the data recorded from both the C- and Aδ-fiber afferents to develop and validate the model. While there are reported differences in the transduction mechanisms between these two afferent groups (Woolf and Ma, 2007; Dubin and Patapoutian, 2010), we did not observe any statistically significant differences in their mechanical response properties and, therefore, did not discriminate between them in the model. Third, the studies identifying the types of proteins expressed on nociceptor membranes are typically performed on cultured DRGs. In contrast, we assumed that the transmembrane proteins expressed on the nerve endings of the muscle nociceptor (which we modeled) are the same as in the DRGs and have similar distributions. While this is a valid assumption, the protein expression on some nociceptive endings might significantly differ from those of the cell body. Fourth, in our model, we adopted many parameter values from previous computational studies developed to describe neurons from animals other than mice or from physiological tissues different from muscle (Mandge and Manchanda, 2018). While we did perform calibration procedures to match our computational simulations to experimental data recorded from sensory neurons innervating the mouse hindpaw muscles, we did not directly derive parameters from single ion channel current measurements in mouse neurons. This simplification could impact the accuracy of certain model parameters. Fifth, while we have incorporated the description of the relevant channels involved in the transduction of mechanical force by the muscle nociceptors, our model does not represent all possible channels and their isomers that are present on the neuronal membrane (Woolf and Ma, 2007; Dubin and Patapoutian, 2010; Mense, 2010). For example, we did not include the thermosensitive channel TRPV1 in the model because we were primarily interested in mechanical nociception. Therefore, there is a possibility that a channel or a specific isomer of a channel currently not included in the model could still be a key regulator of mechanical nociception in muscles. Sixth, our model does not consider the effect of neurotransmitters and inflammatory mediators on the nociceptor response. We have begun work on that front, where we are extending the model to represent the intracellular signaling mechanisms initiated by inflammatory mediators that are released by different cells in the muscle tissue when exposed to noxious stimuli. Finally, our hypotheses regarding the contributions of TRPA1, Kv1.1, and Piezo2 to the response of muscle afferents stem solely from simulations. These hypotheses need to be validated by independent mice experiments, where we separately knockout each protein and assess the effect on AP firing.

## Conclusion

The identification of transmembrane proteins and other signaling molecules in the PNS that regulate the acute pain response to potentially harmful stimuli is a challenge, stemming from the heterogeneity in nociceptor types and functions across different tissues and species. In this study, we specifically focused on nociception in the muscle tissue. To this end, we performed customized experiments, developed a muscle-mechanosensitive neuron-specific computational model, and performed simulations to identify key ion channels that regulate the AP generation in response to mechanical forces. Our results allowed us to hypothesize that Kv1.1, Piezo2, and TRPA1 are the major contributors to AP generation in muscle nociceptors; that KO of Kv1.1 and TRPA1 increases AP firing (i.e., nociceptor excitability); and that these ion channels may play an important role in nociceptor sensitization. Studies to experimentally test our hypotheses will improve the understanding of acute pain initiation in musculoskeletal tissue and help identify signaling alterations that cause nociceptor sensitization.

## Data Availability Statement

The original contributions presented in the study are included in the article/Supplementary Material, further inquiries can be directed to the corresponding author/s.

## Ethics Statement

The animal study was reviewed and approved by Institutional Animal Care and Use Committee (IACUC) at Cincinnati Children’s Hospital Medical Center Animal Care and Use Review Office (ACURO) of the Department of Defense under AAALAC approved practices.

## Author Contributions

SN, MPJ, and JR conceptualized the work. SN developed the model and performed the computational analysis. SGT assisted in the computational analysis. LFQ, MCH, and MPJ designed and performed the experiments. SN and JR wrote the manuscript. All authors reviewed and edited the final manuscript.

## Author Disclaimer

The opinions and assertions contained herein are the private views of the authors and are not to be construed as official or as reflecting the views of the United States Army, the United States Department of Defense, or The Henry M. Jackson Foundation for the Advancement of Military Medicine, Inc. This paper has been approved for public release with unlimited distribution.

## Conflict of Interest

The authors declare that the research was conducted in the absence of any commercial or financial relationships that could be construed as a potential conflict of interest.

## Publisher’s Note

All claims expressed in this article are solely those of the authors and do not necessarily represent those of their affiliated organizations, or those of the publisher, the editors and the reviewers. Any product that may be evaluated in this article, or claim that may be made by its manufacturer, is not guaranteed or endorsed by the publisher.
