# Supplementary material for: In silico Identification of Key Factors Driving the Response of Muscle Sensory Neurons to Noxious Stimuli
Source: Front Neurosci. 2021 Sep 10;15:719735. doi: 10.3389/fnins.2021.719735 (PMC8461020; doi:10.3389/fnins.2021.719735)
Supplement: Supplementary file 1 [file Data_Sheet_1.PDF]

AIC analysis

AIC_c (corrected AIC number) is a single number score that can be used to determine which of multiple models is the most likely to explain the greatest amount of variation in the data using the fewest possible independent parameters. AIC works by evaluating the model's fit on the training data, and adding a penalty term for the complexity (i.e., the number of parameters) of the model. Lower values of AIC indicate a better fit of the model to the data. We calculated the AIC_c value using the formula in Eq. S1.

$$AIC_c = -2ln(L_{max}) + 2P + \frac{2P-(P+1)}{(N-P-1)} \quad \text{(Eq. S1)}$$

where L_{max} denotes the maximum value of the likelihood function defined in Eq. S2, P denotes the number of model parameters, and N denotes the sample size.

$$L_{max}=max\left(\frac{Data_i\ -Sim_i}{Data_{max}\ -Data_{min}}\right) \quad \text{(Eq. S2)}$$

where $Data_i$ denotes the ith value of the data (i.e., #APs fired in response to an applied force of 0.7, 4, and 40 mN) and Sim_i denotes the corresponding simulated value. $Data_{max}$ and $Data_{min}$ represent the maximum and minimum values of the data, respectively.

Table S1. AIC_c values of models with different numbers of parameters used for calibration

Model	# of modified parameters	AIC_c
Nominal	23	-12.11
Nav1.7 added to calibration	25	-12.09
TRPA1 not calibrated	18	-10.30
Piezo1 not calibrated	17	-9.18
Kv1.1 not calibrated	21	-9.97
Kv7.2 not calibrated	22	-8.95
TREK-1 not calibrated	19	-10.05

The results from our AIC analysis in Table S1 showed that in order to calibrate our model to accurately capture the mechanical response characteristics observed in our data, we needed to modify the values of the 23 model parameters associated with five ion channels. When we tried to perform the calibration with fewer parameters, the accuracy of the model fit decreased (as indicated by increasing AIC_c values). Moreover, increasing the number of model parameters used for calibration (e.g., modifying the parameter values associated with Nav1.7 in addition to the 23 parameters) did not improve model accuracy.

Table S2. Baseline and mechanical response characteristics of the afferent neurons recorded in the experiments

Classification	By fiber type		By modality	
	Aδ (N = 13)	C (N = 7)	Unimodal (N = 11)	Polymodal (N = 9)
RMP (mV)	-49.06 (23.4)	-64.04 (11.5)	-59.20 (10.6)	-50.76 (23.1)
AP width (ms)	1.6 (0.8)	2.2 (0.4)	1.74 (0.78)	1.86 (0.73)
AP overshoot (mV)	16.06 (10.4)	17.62 (9.8)	15.20 (10)	17.53 (10)
Mechanical threshold (mN)	44.4 (46.7)	70.0 (38.7)	69.2 0(4.1)	28.60 (4)
Peak AP firing	5.0 (6.2)	3.71 (3.3)	2.27 (1.5)	7.30 (6.5)
Peak instantaneous frequency (Hz)	119.75 (174.3)	35.16 (32.5)	139.50 (183.6)	102.68 (143.2)

Shown are the mean and 1 SD (in parentheses) of the different baseline and mechanical response characteristics of the muscle afferent neurons. We analyzed the data by fiber type (Aδ or C) as well as by modality type (unimodal or polymodal). The unimodal group of afferents was comprised of both Aδ- and C-fiber neurons that only responded to mechanical stimuli, whereas polymodal afferents were comprised of Aδ- and C-fiber neurons that responded to multiple stimuli (i.e., AMCH-Met, CMH-Met, AMC-Met, AM-Met, AMC, CMC)[†]. We used Kruskal-Wallis one-way ANOVA on ranks to compare the characteristics between the two groups. Our critical significance level was set at p<0.05. [*]Significant differences between groups. We did not find any significant differences for any of these characteristics between the two groups.
[†]A: Aδ-fiber, C: C-fiber, M: responded to mechanical stimuli, C: responded to a cold stimulus, H: responded to a heat stimulus, Met: responded to metabolites.

Table S3. Response characteristics of mechanoreceptors vs. mechanical nociceptors

	Type of muscle afferent	
	Mechanoreceptors (N = 5)	Mechanical nociceptors (N = 10)
Mechanical threshold (mN)	18.94 (19.50)	56.47 (47.05)
Peak AP firing	3.20 (1.64)	7.00 (6.59)
AUC for AP firing under all forces	226.73	444.09

Shown are the mean and 1 SD (in parentheses) of the different baseline and mechanical response characteristics of the muscle afferents. We classified our data from 15 mechanically sensitive afferent neurons as mechanical nociceptors or mechanoreceptors. An afferent neuron was classified as a mechanical nociceptor based on its ability to encode the severity of the mechanical force input in the AP response. In addition, an afferent neuron that fired APs for forces >20mN was considered a nociceptor. We used Kruskal-Wallis one-way ANOVA on ranks to compare the characteristics between the two groups. Our critical significance level was set at p<0.05.
[*]Significant differences between groups.

Model equations for description of transmembrane currents, endoplasmic reticulum (ER) mechanisms, Nernst potentials, and balance of intracellular Na^+, K^+, and Ca^{2+} ions.

Transmembrane mechanisms

1. Voltage-gated Nav1.8 channel

$$\frac{\mathrm{dNav1.8_m}}{\mathrm{d}t}=\frac{\mathrm{-Nav1.8_m+Nav1.8_{mss}}}{\tau_{\mathrm{m_{Nav1.8}}}}$$

$$\frac{\mathrm{dNav1.8_h}}{\mathrm{d}t}=\frac{\mathrm{-Nav1.8_h+Nav1.8_{hss}}}{\tau_{\mathrm{h_{Nav1.8}}}}$$

$$\mathrm{am_r}=\frac{7.2}{1+e^{((V_\mathrm{m}-0.063)/7.86)}}$$

$$\mathrm{bm_r}=\frac{7.4}{1+e^{((V_\mathrm{m}+53.06)/19.34)}}$$

$$\mathrm{ah_r}=0.003+\frac{1.63}{1+e^{((V_\mathrm{m}+68.5)/10.01)}}$$

$$\mathrm{bh_r}=0.81-\frac{0.81}{1+e^{((V_\mathrm{m}-11.44)/13.12)}}$$

$$\tau_{\mathrm{m_{Nav1.8}}}=\frac{1}{\mathrm{am_r+bm_r}}$$

$$\tau_{\mathrm{h_{Nav1.8}}}=\frac{1}{\mathrm{ah_r+bh_r}}$$

$$\mathrm{Nav1.8_{mss}}=\frac{1}{1+e^{\left(\frac{\mathrm{Vm_{Nav1.8}}-V_\mathrm{m}}{\mathrm{kact_{Nav1.8}}}\right)}}$$

$$\mathrm{Nav1.8_{hss}}=\frac{1}{1+e^{\left(\frac{V_\mathrm{m}+\mathrm{Vh_{Nav1.8}}}{\mathrm{kinact_{Nav1.8}}}\right)}}$$

$$I_\mathrm{Nav1.8}=\mathrm{Imax_{Nav1.8}}\cdot\mathrm{Nav1.8_m^2}\cdot\mathrm{Nav1.8_h}\cdot(V_\mathrm{m}-V_\mathrm{Na})$$

2. Voltage-gated Nav1.9 channel

$$\frac{\mathrm{dNav1.9_m}}{\mathrm{d}t}=\frac{\mathrm{-Nav1.9_m+Nav1.9_{mss}}}{\tau_{\mathrm{m_{Nav1.9}}}}$$

$$\frac{\mathrm{dNav1.9_h}}{\mathrm{d}t}=\frac{\mathrm{-Nav1.9_h+Nav1.9_{hss}}}{\tau_{\mathrm{h_{Nav1.9}}}}$$

$$am_{1.9}=\frac{1.548}{1+e^{((V_m-11.01)/-14.871)}}$$

$$bm_{1.9}=\frac{8.685}{1+e^{((V_m+112.4)/22.9)}}$$

$$ah_{1.9}=\frac{0.2574}{1+e^{((V_m+63.264)/3.719)}}$$

$$bh_{1.9}=\frac{0.54}{1+e^{((V_m+0.28)/-0.093)}}$$

$$\tau_{m_{Nav1.9}}=\frac{1}{am_{1.9}+bm_{1.9}}$$

$$\tau_{h_{Nav1.9}}=\frac{1}{ah_{1.9}+bh_{1.9}}$$

$$Nav1.9_{mss}=\frac{am_{1.9}}{am_{1.9}+bm_{1.9}}$$

$$Nav1.9_{hss}=\frac{ah_{1.9}}{ah_{1.9}+bh_{1.9}}$$

$$I_{Nav1.9}= Imax_{Nav1.9}\cdot Nav1.9_m^2\cdot Nav1.9_h\cdot(V_m-V_{Na})$$

3. Voltage-gated Nav1.7 channel

$$\frac{dNav1.7_m}{dt}=\frac{-Nav1.7_m+Nav1.7_{mss}}{\tau_{m_{Nav1.7}}}$$

$$\frac{dNav1.7_h}{dt}=\frac{-Nav1.7_h+Nav1.7_{hss}}{\tau_{h_{Nav1.7}}}$$

$$am_{1.7}=\frac{15.5}{1+e^{((V_m-5)/-12.08)}}$$

$$bm_{1.7}=\frac{35.2}{1+e^{((V_m+72.7)/16.7)}}$$

$$ah_{1.7}=0.24\cdot e^{(-\frac{V_m+115}{46.33})}$$

$$bh_{1.7}=4.32\cdot(1+e^{(\frac{V_m-11.8}{-12})})$$

$$\tau_{m_{Nav1.7}}=\frac{1}{am_{1.7}+bm_{1.7}}$$

$$\tau_{h_{Nav1.7}}=\frac{1}{ah_{1.7}+bh_{1.7}}$$

$$\mathrm{Nav1.7_{mss}}=\frac{1}{1+e^{(\frac{\mathrm{Vm_{Nav1.7}}-V_m}{\mathrm{kact_{Nav1.7}}})}}$$

$$\mathrm{Nav1.7_{hss}}=\frac{1}{1+e^{(\frac{V_m+\mathrm{Vh_{Nav1.7}}}{\mathrm{kinact_{Nav1.7}}})}}$$

$$I_{\mathrm{Nav1.7}}=\mathrm{Imax_{Nav1.7}}\cdot\mathrm{Nav1.7_m^2}\cdot\mathrm{Nav1.7_h}\cdot(V_m-V_{\mathrm{Na}})$$

4. Mechanosensitive Piezo2 channel

$$\frac{\mathrm{dPiezo_m}}{\mathrm{d}t}=\frac{\mathrm{Piezo_m}+\mathrm{Piezo_{mss}}}{\mathrm{\tau act_{Piezo}}}$$

$$\frac{\mathrm{dPiezo_h}}{\mathrm{d}t}=\frac{\mathrm{Piezo_h}+\mathrm{Piezo_{hss}}}{\mathrm{\tau inact_{Piezo}}}$$

$$\mathrm{Piezo_{mss}}=\frac{1}{1+e^{(\frac{\mathrm{Vm_{Piezo}}-\mathrm{Mechforce}}{\mathrm{kact_{Piezo}}})}}$$

$$\mathrm{Piezo_{hss}}=1-\frac{1}{1+e^{(\frac{\mathrm{Vh_{Piezo}}-\mathrm{Mechforce}}{\mathrm{kinact_{Piezo}}})}}$$

$$I_{\mathrm{PiezoNa}}=\mathrm{Imax_{Piezo}}\cdot\mathrm{Piezo_m^4}\cdot\mathrm{Piezo_h^2}\cdot(V_m-V_{\mathrm{Na}})$$

$$I_{\mathrm{PiezoCa}}=\mathrm{Imax_{Piezo}}\cdot\mathrm{Piezo_m^4}\cdot\mathrm{Piezo_h^2}\cdot(V_m-V_{\mathrm{Ca}})$$

$$I_{\mathrm{Piezo}}=I_{\mathrm{PiezoNa}}+I_{\mathrm{PiezoCa}}$$

5. Mechanosensitive TRPA1 channel

$$\frac{\mathrm{dTRPA1_m}}{\mathrm{d}t}=\frac{-\mathrm{TRPA1_m}+\mathrm{TRPA1_{mss}}}{\mathrm{\tau act_{TRPA1}}}$$

$$\frac{\mathrm{dTRPA1_h}}{\mathrm{d}t}=\frac{-\mathrm{TRPA1_h}+\mathrm{TRPA1_{hss}}}{\mathrm{\tau inact_{TRPA1}}}$$

$$\mathrm{TRPA1_{mss}}=\frac{1}{1+e^{(\frac{\mathrm{Vm_{TRPA1}}-\mathrm{Mechforce}}{\mathrm{kact_{TRPA1}}})}}$$

$$\mathrm{TRPA1_{hss}}=1-\frac{1}{1+e^{(\frac{\mathrm{Vh_{TRPA1}}-\mathrm{Mechforce}}{\mathrm{kinact_{TRPA1}}})}}$$

$$I_{\mathrm{TRPA1}}=\mathrm{Imax_{TRPA1}}\cdot\mathrm{TRPA1_m^2}\cdot\mathrm{TRPA1_h}\cdot(V_m-V_{\mathrm{Na}})$$

6. Mechanosensitive two-pore TREK-1 channel

$$\frac{dTREK_m}{dt}=\frac{-TREK_m+TREK_{mss}}{\tau act_{TREK}}$$

$$TREK_{mss}=\frac{1}{1+e^{(\frac{Vm_{TREK}-Mechforce}{kact_{TREK}})}}$$

$$I_{TREK1}= Imax_{TREK}\cdot TREK_m^2\cdot(V_m-V_K)$$

Mechforce = 0.7, 4, 10, 20, 40, or 100

7. pH-mediated ASIC3 channel

$$\frac{dASIC3_m}{dt}=\frac{-ASIC3_m+ASIC3_{mss}}{\tau act_{ASIC3}}$$

$$\frac{dASIC3_h}{dt}=\frac{-ASIC3_h+ASIC3_{hss}}{\tau inact_{ASIC3}}$$

$$\tau inact_{ASIC3}=197.36\cdot pH^2 - 1738.9\cdot pH + 3968.1$$

$$ASIC3_{mss}=\frac{1}{1+e^{(\frac{Vm_{ASIC3}-pH}{kact_{ASIC3}})}}$$

$$ASIC3_{hss}=1-\frac{1}{1+e^{(\frac{Vh_{ASIC3}-pH}{kinact_{ASIC3}})}}$$

$$I_{ASIC3}= Imax_{ASIC3}\cdot ASIC3_m\cdot ASIC3_h\cdot(V_m-V_{Na})$$

pH = 7.5

8. Voltage-gated Kv7.2 channel

$$\frac{dKv7_n}{dt}=\frac{-Kv7_n+Kv7_{nss}}{\tau_{n_{Kv7}}}$$

$$an_{Kv7}=k1act_{Kv7}\cdot e^{(\frac{V_m+Vm_{Kv7}}{k2act_{Kv7}})}$$

$$bn_{Kv7}=k1inact_{Kv7}\cdot e^{-(\frac{V_m+Vm_{Kv7}}{k2inact_{Kv7}})}$$

$$\tau_{n_{Kv7}}=\frac{1}{an_{Kv7}+bn_{Kv7}}$$

$$Kv7_{nss}=\frac{1}{1+e^{(\frac{-V_m-Vm_{Kv7}}{\tau act_{Kv7}})}}$$

$$I_{Kv7.2} = \mathrm{Imax}_{Kv7} \cdot \mathrm{Kv7}_n^2 \cdot (V_m - V_K)$$

9. Delayed-rectifier Kv1.1 K^+ channel

$$\frac{d\mathrm{Kv1.1}_n}{dt} = \frac{-\mathrm{Kv1.1}_n + \mathrm{Kv1.1}_{nss}}{\tau act_{Kv1.1}}$$

$$\mathrm{Kv1.1}_{nss} = \frac{\delta_{Kv1.1}}{1 + e^{\left(\frac{Vm_{Kv1.1} - V_m}{kact_{Kv1.1}}\right)}}$$

$$I_{Kv1.1} = \mathrm{Imax}_{Kv1.1} \cdot \mathrm{Kv1.1}_n^2 \cdot (V_m - V_K)$$

10. Voltage-gated A-type K^+ channel

$$\frac{d\mathrm{Ka}_n}{dt} = \frac{-\mathrm{Ka}_n + \mathrm{Ka}_{nss}}{\tau_{n_{Ka}}}$$

$$\frac{d\mathrm{Ka}_{hfast}}{dt} = \frac{-\mathrm{Ka}_{hfast} + \mathrm{Ka}_{hfastss}}{\tau_{hfast_{Ka}}}$$

$$\frac{d\mathrm{Ka}_{hslow}}{dt} = \frac{-\mathrm{Ka}_{hslow} + \mathrm{Ka}_{hslowss}}{\tau_{hslow_{Ka}}}$$

$$\mathrm{Ka}_{nss} = \frac{1}{1 + e^{\left(\frac{-V_m - 40.8}{9.5}\right)}}$$

$$\mathrm{Ka}_{hfastss} = \frac{1}{1 + e^{\left(\frac{V_m + 74.2}{9.6}\right)}}$$

$$\tau_{n_{Ka}} = 1.2 + 2.56 \cdot e^{\left(-2 \cdot \left(\frac{V_m + 60}{45.768}\right)^2\right)}$$

$$\tau_{hfast_{Ka}} = 25.46 + 67.41 \cdot e^{\left(-2 \cdot \left(\frac{V_m + 50}{21.95}\right)^2\right)}$$

$$\tau_{hslow_{Ka}} = 200 + 587.4 \cdot e^{\left(-\left(\frac{V_m}{47.77}\right)^2\right)}$$

$$I_{Ka} = \mathrm{Imax}_{Ka} \cdot \mathrm{Ka}_n \cdot (0.3\mathrm{Ka}_{hfast} + 0.7\mathrm{Ka}_{hslow}) \cdot (V_m - V_K)$$

11. Large-conductance Ca^{2+}-activated K^+ channel

$$\frac{d\mathrm{BKCa}_n}{dt} = \frac{-\mathrm{BKCa}_n + \mathrm{BKCa}_{nss}}{\tau_{n_{BKCa}}}$$

$$p_{Ca} = \log_{10} \cdot (\mathrm{Ca}_i e^{-3})$$

$kact_{BKCa} = (-43.4 \cdot p_{Ca}) - 203$

$sf_{BKCa} = 33.88 \cdot e^{-(p_{Ca}+5.42)/1.85^2}$

$$BKCa_{nss} = \frac{1}{1+e^{(\frac{kact_{BKCa}-V_m}{sf_{BKCa}})}}$$

$$\tau_{n_{BKCa}} = 5.55 \cdot e^{\frac{V_m}{42.91}} + 0.75 - (0.12 \cdot V_m)$$

$$I_{BKCa} = Imax_{BKCa} \cdot BKCa_n^2 \cdot (V_m - V_K)$$

12. T-type voltage-gated Ca^{2+} channel

$$\frac{dCaT_m}{dt} = \frac{-CaT_m + CaT_{mss}}{\tau act_{CaT}}$$

$$\frac{dCaT_h}{dt} = \frac{-CaT_h + CaT_{hss}}{\tau inact_{CaT}}$$

$$CaT_{mss} = \frac{1}{1+e^{(\frac{V_m - Vm_{CaT}}{kact_{CaT}})}}$$

$$CaT_{hss} = 1 - \frac{1}{1+e^{(\frac{V_m - Vh_{CaT}}{kinact_{CaT}})}}$$

$$I_{CaT} = Imax_{CaT} \cdot CaT_m^2 \cdot CaT_h \cdot (V_m - V_{Ca})$$

13. L-type voltage-gated Ca^{2+} channel

$$\frac{dCaL_m}{dt} = \frac{-CaL_m + CaL_{mss}}{\tau act_{CaL}}$$

$$\frac{dCaL_h}{dt} = \frac{-CaL_h + CaL_{hss}}{\tau inact_{CaL}}$$

$$CaL_{mss} = \frac{1}{1+e^{(\frac{Vm_{CaL} - V_m}{kact_{CaL}})}}$$

$$CaL_{hss} = 1 - \frac{1}{1+e^{(\frac{Vh_{CaL} - V_m}{kinact_{CaL}})}}$$

$$hCa_{CaL} = \frac{1}{1+(Ca_i/1e^{-6})^4}$$

$$I_{CaL} = Imax_{CaL} \cdot CaL_m \cdot CaL_h \cdot hCa_{CaL} \cdot (V_m - V_{Ca})$$

14. NaK pump

$$I_{\mathrm{NaK}}=\mathrm{Imax}_{\mathrm{NaK}}\cdot\frac{\mathrm{K}_o^2}{\mathrm{K}_o^2+\mathrm{KK}_{\mathrm{NaK}}^2}\cdot\frac{\mathrm{Na}_i^{\mathrm{nH}_{\mathrm{Na}}}}{\mathrm{Na}_i^{\mathrm{nH}_{\mathrm{Na}}}+\mathrm{KNa}_{\mathrm{NaK}}^{\mathrm{nH}_{\mathrm{Na}}}}\cdot\frac{V_m+70}{V_m+180}$$

15. PMCA pump

$$I_{\mathrm{PMCA}}=\mathrm{Imax}_{\mathrm{PMCA}}\cdot\frac{\mathrm{Ca}_i}{\mathrm{Ca}_i+\mathrm{KCa}_{\mathrm{PMCA}}}$$

16. Na^{+}-Ca^{2+} exchanger

$$\mathrm{kqa}=e^{\frac{0.35\cdot V_m}{k_{\mathrm{NCX}}}}$$

$$\mathrm{kb}_{\mathrm{NCX}}=e^{\frac{-0.65\cdot V_m}{k_{\mathrm{NCX}}}}$$

$$I_{\mathrm{NCX}}=\mathrm{Imax}_{\mathrm{NCX}}\cdot\ (\mathrm{kqa}\cdot\mathrm{Na}_i^3\cdot\mathrm{Ca}_o)-\frac{\mathrm{kb}_{\mathrm{NCX}}\cdot\mathrm{Ca}_i\cdot\mathrm{Na}_o^3}{(\mathrm{kNa}^3+\mathrm{Na}_o^3)\cdot(\mathrm{kCa}+\mathrm{Ca}_o)\cdot(1+0.1\mathrm{kb}_{\mathrm{NCX}})}$$

17. Passive K^{+} leak channel

$$I_{\mathrm{Kleak}}=\mathrm{Imax}_{\mathrm{Kleak}}\cdot(V_m-(-45))$$

ER mechanisms

1. IP_3 receptor flux

$$\frac{\mathrm{dhIP}_3}{\mathrm{d}t}=\mathrm{kf}_{\mathrm{IP3}}\cdot(\mathrm{kb}_{\mathrm{IP3}}-(\mathrm{Ca}_i+\mathrm{kb}_{\mathrm{IP3}})\cdot\mathrm{hIP}_3)$$

$$I_{\mathrm{IP3R}}=\mathrm{Imax}_{\mathrm{IP3R}}\cdot\left(\frac{\mathrm{IP}_3}{\mathrm{IP}_3+\mathrm{k}_{\mathrm{IP3}}}\right)\cdot\left(\frac{\mathrm{Ca}_i}{\mathrm{Ca}_i+\mathrm{kCa}_{\mathrm{IP3}}}\cdot\mathrm{hIP}_3\right)^3\cdot\left(1-\frac{\mathrm{Ca}_i}{\mathrm{Ca}_{\mathrm{ER}}}\right)$$

2. SERCA pump

$$I_{\mathrm{SERCA}}=\mathrm{Imax}_{\mathrm{SERCA}}\cdot\left(\frac{\mathrm{Ca}_i^2}{\mathrm{Ca}_i^2+\mathrm{KCa}_{\mathrm{SERCA}}^2}\right)$$

3. ER leak current

$$I_{\mathrm{leakER}}=5\times10^{-7}\cdot\left(1-\frac{\mathrm{Ca}_i}{\mathrm{Ca}_{\mathrm{ER}}}\right)$$

$I_{\text{leakER}} = \text{Imax}_{\text{ERleak}} \cdot \left(1 - \frac{\text{Ca}_i}{\text{Ca}_{\text{ER}}}\right)$ if $\text{Ca}_i > \text{KTCa}$

4. Ryanodine receptor flux

$I_{\text{CICR}} = \text{Imax}_{\text{CICR}} \cdot \left(\frac{\text{Ca}_i}{\text{Ca}_i + \text{KCa}_{\text{CICR}}}\right) \cdot (\text{Ca}_{\text{ER}} - \text{Ca}_i)$ if $\text{Ca}_i > \text{KTCa}$

$I_{\text{CICR}} = 0$

5. Ca_i^{2+} buffering in cytosol and ER

$$\beta_{\text{ER}} = \frac{\text{CSQN} \cdot \text{K}_{\text{CSQN}}}{(\text{K}_{\text{CSQN}} + \text{Ca}_{\text{ER}})^2}$$

Nernst potential calculations

$$V_{\text{Na}} = \frac{\text{R} \cdot \text{T}}{\text{zNa} \cdot \text{F}} \cdot \log\left(\frac{\text{Na}_o}{\text{Na}_i}\right)$$

$$V_{\text{K}} = \frac{\text{R} \cdot \text{T}}{\text{zK} \cdot \text{F}} \cdot \log\left(\frac{\text{K}_o}{\text{K}_i}\right)$$

$$V_{\text{Ca}} = \frac{\text{R} \cdot \text{T}}{\text{zCa} \cdot \text{F}} \cdot \log\left(\frac{\text{Ca}_o}{\text{Ca}_i}\right)$$

Ionic balances

$$\frac{d\text{Ca}_i}{dt} = -(I_{\text{CaT}} + I_{\text{CaL}} + I_{\text{PMCA}} - 2I_{\text{NCX}} + I_{\text{PiezoCa}})/(\text{zCa} \cdot \text{F} \cdot 0.7 \cdot \text{vol}) - (I_{\text{SERCA}} - I_{\text{leakER}} - I_{\text{IP3}} - I_{\text{CICR}})/(1/(1+370))$$

$$\frac{d\text{Ca}_{\text{ER}}}{dt} = I_{\text{SERCA}} - I_{\text{leakER}} - I_{\text{IP3}} - I_{\text{CICR}} / \beta_{ER}$$

$$\frac{d\text{Na}_i}{dt} = -(I_{\text{Nav1.8}} + I_{\text{Nav1.9}} + I_{\text{Nav1.7}} + I_{\text{PiezoNa}} + I_{\text{TRPA1}} + 3I_{\text{NaK}} + 3I_{\text{NCX}})/(\text{zNa} \cdot \text{F} \cdot \text{vol})$$

$$\frac{d\text{K}_i}{dt} = -(I_{\text{TREK1}} + I_{\text{Kv7.2}} + I_{\text{Kv1.1}} + I_{\text{BKCa}} + I_{\text{Ka}} + I_{\text{Kleak}} - 2I_{\text{NaK}})/(\text{zK} \cdot \text{F} \cdot \text{vol})$$
